# 

**Published:** 1888-01

**Authors:** 


### INDEX TO VOLUME XLII.

Ammonia vs. Zinc Fillings..... 25
Abscess of the Lung Probably Caused by the Stump of a Tooth Passing into the Right Bronchus............... 92
Antiseptic Mouth-Wash.......... 152
American Med. Association.155, 314
Address of President W. N. Morrison......................... 161
Antiseptics in the Mouth......... 194
A New Antiseptic.................. 204
Aetiology of Irregularities of the Jaws and Teeth.......... 213
A Case in Office Practice.......... 238
Aluminum Cast Dentures......... 294
Aluminum Cast Dentures, Discussion on......................... 295
A Rescued Fragment............... 300
Address — Atkinson — Indiana College ......................... 317
Art in Dentistry...................... 336
American and Southern Dental Associations ..................... 365
A Cheap Battery...................... 411
A Correction, Inter. Med. Cong. 412
Anæsthetics in Dental Practice 421
Artificial Vula for Congenital Cleft Palate Case.............. 435
Asphalte Pavements and Public Health............................ 458
A Practical Disinfectant—Corrosive Sublimate............... 464
Anæsthetics in Dental Practice 473
A Centinarian......................... 511
Address — Catching — Before Am. and So. Associations 525
Amer. and So. Dental Associations Joint Meeting........... 531
Breathing Pure Oxygen........... 17
Boracine.................................. 103
Bibliographical—Dental Metallurgy—Essay.................... 210
Bibliographical—Dentists' Manual of Special Chemistry—Mitchell........................... 211
Bibliographical— Irregularities of the Teeth and their Treatment—Talbot........... 264
Book Notice............................. 315
Brain — Corrosive Sublimate for Hardening.................. 413
Bone Repair—the Study—Miller....................................... 443
Cleveland Dental Society......... 54
Crown and Bridge Work, Legal Status of........................... 99
Correspondence........................ 102
Correspondence—A New Tooth Crown.............................. 105
Correspondence—Indiana Dental Examiners................... 154
Correction............................... 159
Crowns—Porcelain—Moore...... 285
Correspondence—Buzzell.......... 357
Correspondence—Beadles—...... 361
Cancrum or Scirrhus Gangrenousa of Inferior Maxillary 369
Correspondence—Disfigured for Life—Corbin..................... 415
California State Dental Society 420
Cocaine in Surgery—Hænel ..... 449
Correspondence—Mitchell........ 466
Correspondence—Cox............... 564
Discussions of the Ohio State Dental Society.................. 42
Dr. N. S. Hoff.......................... 54
Diseased Gums......................... 104
Dental Irritation as a Factor in the Cousation of Epilepsy... 138
Duty on Medical Supplies........ 148
Dental Pulp—My Experience with, W. A. Pease............. 164
Dental Pulp, J. E. Cravens....... 175
Dental Pulp, Conservation of.... 177
Dental Pulps, Gangrenous, as Centres of Infection........... 180
Dentogeny—Brittan................ 265
Dentogeny — Discussion of—Fletcher ........................ 280
Diseases that Affect the Oral Structures—Martin........... 349
Dental Colleges, Lists of Graduates ............................. 362
Development of the Teeth—Nicol.................................. 373

Dental Faculties Association—Annual Meeting..........418, 497
Delayed Dentition.................. 502
Dental Examinations, National Association of..................
Dental Caries and Reflex Paralysis.................................. 506
Distilled Water....................... 570
Dentists' Register..................... 576
Extraction of Teeth................. 32
English as Written.................. 54
Editorial—Progress in Anæsthesia.................................... 157
Editorial—Amer. Med. Asso..... 208
Editorial—Obituary................ 263
Editorial—Educational............ 313
Electricity in Surgery—Maclean 398
Editorial—National Asso. of Dental Examiners............. 416
Editorial—Obituary ............... 417
Editorial—Foggots, Corundum, Point and Disc Maker........ 470
Editorial—A Desiccating Instrument................................ 471
Editorial—Obituary—Keely..... 472
Examination of the Teeth for Bacteria.............................. 477
Extraction of a Tooth, Reflex Pain Following................. 505
Early Rising and Longevity..... 511
Eye Inflammation................... 571
Editorial—Dinner to Dr. McKellops.............................. 574
Editorial—Dentists' Register.... 576
Editorial—Dental Colleges...... 596
First District Dental Society of New York......................... 50
For Chapped Hands............... 94
Floors, Cracks in..................... 99
First Permanent Molar, Monograph of............................. 120
First Permanent Molar, C. S. Case.................................. 340
Georgia State Dental Society Meeting............................. 482
Hæmoptysis—Case of ............ 147
Heredity, Influence of upon Deformities and Defects......... 150
How to Clean the Hands.......... 204
How to Tell a Horse's Age....... 509
In Memoriam—Dr. A. B. Palmer 55
Inflammation about the Third Molar................................ 109
Implantation—Young's Operation.................................... 152
Intermittent Pressure—Its Relation to Orthodontia........... 169
Implantation with Clinical Demonstrations................ 233
Immediate Root Filling........... 242
Iodoform, Deodorants for......... 293
Illinois State Dental Society—Annual Meeting.............. 377
Information.......................... 419
Iodoform Paste...................... 592
Kentucky State Dental Society, Annual Meeting.............. 393
Lupus and Cutaneous Tuberculosis.................................. 460
Mouth Wash......................... 94
Mississippi Valley Dental Association ............................. 102
Mumphs, Incision for............. 105
Mississippi Valley Association of Dental Surgeons........... 107
Michigan State Dental Society.. 159
Medical Editors, Meeting of..... 212
Medical Missourians.............. 259
Mad River Dental Associations. 366
Mucous Membrane—Transplantation of......................... 412
Microscopy.......................... 455
Near Approach to and Devitalization of the Dental Pulp. 23
Ninth International Med. Congress XVIIIth Section, Dental and Oral Surgery......... 84
Neuralgia Facial Treatment of, by Antipyrine.................. 98
Neuralgia—Belladonna and Nux Vomica........................... 103
Neuralgia, Remedy................ 104
Naphthol as an Antiseptic........ 149
New Blistering Liquid............ 153
Neuralgia, Chloroform and the Constant Current in.......... 205
Neuralgia, Contribution to the Etiology of...................... 258
Non-Union of Bone During Pregnancy ......................... 310
Nerve Transplantation from Animals to Man............... 344
Obituary—Dr. J. M. Prewitt..... 108
Orthodontia—Dr. N. S. Hoff..... 115
Obituary—Dunster................. 316
Oral Antisepsis...................... 327

Oral Antisepsis, Discussion of... 331
Over Population of Towns........ 459
Oil of Peppermint as an Antiseptic............................ 507
Obituary—Driggs..................... 518
Ohio District Dental Societies... 595
Protective Dentine or Dentine of Repair........................ 5
Pyorrhæa Alveolaris................ 35
Painless Tooth Extraction........ 104
Photographic Diagnosis........... 149
Pure Food Convention............. 151
Photography in Surgery........... 201
Proceedings of Mississippi Valley Dental Association....... 245
Practical Application of Electrolysis.......................... 333
Pyorrhœa Alveolits in the Elephant.............................. 454
Peculiarities of American Eyes. 570
Reflex—(ions)........................ 27
Reasons for Using Heavy Gold Foil in Filling Teeth......... 59
Rubber in Prosthetic Dentistry, R. Russell....................... 131
Removal—Dr. Bogne............... 160
Some Suggestions on Metal Cap Crowns.......................... 17
Southern Dental Association Nineteenth Annual Session. 63
Salmagundi, 205, 261, 311, 358, 413, 521
Stockholders Meeting — Ohio College Dental Surgery...... 250
Scurvy—Three Cases of............ 401
Saliva—Berry........................ 432
Tin and Gold as a Filling......... 31
The Nose and the Anterior Passages in Respiration.......... 95
Teeth in Inherited Syphilis...... 150
Teeth in Congenital Syphilis.... 153
Tin and Gold Filling—Remarks on.................................. 186
Tumors — Non-Malignant and Malignant — Differential Points............................ 192
The Six-Year Molars............... 367
The Tongue — Amputation of Half—McClellan............... 408
Tin and Gold Filling—Miller... 441
Tetanus-Three Recoveries from. 456
The Time in which We Think... 461
Treatment of Ulcers................ 508
The Spread of Antisepsis......... 514
The Nails—Care of.................. 524
To Solder Steel....................... 572
Toothache—Solidified Creosote. 573
What Medical Treatment is often Required in Connection with Filling Teeth?.......... 59
What Cocaine to Use................ 508
Who is my Neighbor............... 513

### First District Dental Society of New York.

The Nineteenth Annaversary of this body will be held in New York City on the 16th, 17th, and 18th of January prox. Preparations are being made for a large and interesting meeting. Those who have attended the meetings of this society on former occasions need not be told what to expect, and especially when they understand that preparations are being made for a grander meeting than ever before. Of the records of this body its members may well be proud, and in many respects it sets a noble example to other societies. Below is given the full programme.

Masonic Temple, Twenty-third street and Central Avenue, January 16, 17, and 18, 1888.

PROGRAMME.

*Monday Evening, January 16th*, 1888.

*Prayer*, . . . BRADY E. BACKUS, D.D., New York.
*Address of Welcome*, DR. WM. WALLACE WALKER, New York.
*Response*, . . . DR. LOUIS JACK, Philadelphia, Pa.

PAPERS.

PAST AND FUTURE LITIGATION AFFECTING DENTISTRY, J. KIMBERLY BEACH ESQ., New Haven, Conn.

L. D. SHEPARD, D.M.D., . . . . Boston, Mass.
Subject: "Prominence of Discussion upon One Tooth to Be Deprecated."

*Tuesday Afternoon, January 17th*, 2.30 *o'clock*.

PAPER.

G. V. BLACK, D.M, D.D.S., . . . Jacksonville, Ill.
Subject: "An Examination of the Disturbing Forces in Physics, with Relation to the Germ Theory."
Discussion opened by
W. ZAVIER SUDDUTH, M.D., D.D.S., . Philadelphia, Pa.
Followed by
W. C. BARRETT, M.D., D.D.S., . . Buffalo, New York.
C. T. STOCKWELL, D.D.S., . . . Springfield, Mass.

*Tuesday Evening, January 17th.*

PAPER.

Frank Abbott, M.D., . . . . . . New York.

Subject: "A Contribution to the Knowledge of Tumors of the Jaws."—Carl Heitzmann, M.D., Frank Abbott, M.D.

Discussion opened by

R. R. Andrews, D.D.S., . . . . Cambridge, Mass.

Followed by

C. Heitzmann, M.D., . . . . . . New York.
W. Zavier Sudduth, M.D., D.D.S., . . Philadelphia, Pa.
Truman W. Brophy, M.D., D.D.S., . . . Chicago, Ill.

*Wednesday Afternoon, January 18th, 2.30o'clock.*

PAPER.

J. N. Farrar, M.D., D.D.S., . . . . . New York.

Subject: "Phylosophy of Correcting Irregularities of the Teeth."

Discussion opened by

S. H. Guilford, A.M., D.D.S., . . . Philadelphia, Pa.

Followed by

E. S. Talbot, D.D.S., . . . . . . Chicago, Ill.
B. S. Byrnes, D.D.S., . . . . . . Memphis, Tenn.
V. H. Jackson, M.D., D.D.S., . . . . . New York.
Norman W. Kingsley, D.D.S., . . . . . New York.

*Wednesday Evening, January 18th.*

PAPER.

Wm. H. Atkinson, M.D., D.D.S., . . . . New York.

Subject: "Studies of Pyorrhea Alveolaris, with Illustrations from a Six Week's Kitten."

Discussion opened by

A. W. Harlan, D.D.S., M. D., . . . . Chicago, Ill.

Followed by

N. C. Pierce, D.D.S, . . . . . . Philadelphia, Pa.
Edwin T. Darby, D.D.S., . . . . Philadelphia, Pa.
James Turman, D.D.S., . . . . . Philadelphia, Pa.
R. B. Adair, D.D.S., . . . . . . Gainsville, Ga.
M. L. Rhein, M.D., D.D.S., . . . . . New York.
A. R. Starr, M.D., D.D.S., . . . . . . New York.
J. N. Farrar, M.D., D.D.S. . . . . New York.

### CLINICS.

WILL BE HELD AT THE

### *NEW YORK COLLEGE OF DENTISTRY,*

SECOND AVENUE AND 23D STREET.

Clinics Commence at 9 A. M., and Last till 12 o'clock Sharp.

*Tuesday, January 17th.*

E. C. KIRK, D.D.S., . . . . Philadelphia, Pa.
Implantation.

R. B. ADAIR, D.D.S., . . . . . . Gainsville, Ga.
Filling Simple Proximal Cavities in Incisors, Using Non-Cohesive Gold Foil.

J. BOND LITTIG, D.D.S., . . . . . . New York.
Contouring Front Teeth with Porcelain.

G. H. WINKLER, M.D., D.D.S., . . . Augusta, Ga.
Immediate Extirpation of the Pulp, and Filling Pulp Cavity.

W. C. WARDLAW, D.D.S., . . . . . Augusta, Ga.
Filling Approximal Cavities of Lower Teeth with a Combination of Gold and Tin.

A. W. HARLAN, M.D., D.D.S., . . . Chicago, Ill.
Treatment of Pyorrhea Alveolaris.

T. T. MOORE, D.D.S., . . . . . Columbia, S. C.
Combination Non-Cohesive and Cohesive Gold Filling.

DR. C. FRANK BLIVIN, . . . . . Worcester, Mass.
An Experiment in Pneumatics Demonstrating its Practical Success.

T. S. WATERS, D.D.S., . . . . . Baltimore, Md.
Removable Bridge.

R. B. ADAIR, D.D.S., . . . . . . Gainsville, Ga.
Treatment of Pyorrhea Alveolaris.

A. G. BENNETT, D.D.S., . . . . . Philadelphia, Pa.
Crown and Bridge Work.

C. N. PIERCE, D.D.S., . . . . . Philadelphia, Pa.
Filling with Soft Gold Foil Under Water.

W. G. A. BRONWILL, D.D.S., . . . . Philadelphia, Pa.
Demonstrating Use of Bonwill Mechanical Mallet.

FRANK ABBOTT, M.D., . . . . . . New York.

Demonstrating Use of Abbott Automatic Mallet.

H. C. Register, M.D., D.D.S., . . . . Philadelphia, Pa.

Treatment of Pulpless Teeth, Filling Root and Crown Cavity at Same Sitting. Also Demonstrating Use of Register's Mechanical Mallet.

J. F. P. Hodson, D.D.S., . . . . . . New York.

Filling Teeth, Using Watt's Crystal Gold.

R. I. Verplanck, D.D.S., . . . . . . Albany.

Will Fill Teeth, Using the Verplanck Matrix and Matrix Pluggers.

Dr. E. S. Gaylord, . . . . . New Haven, Conn.

Will demonstrate the use of his Ivory-pointed Pluggers. Also a new Rubber Dam-Holder.

A. H. Gilroy, D.D.S., . . . . . . Boston, Mass.

Will demonstrate his Pneumatic Mallet Hand-piece and Chip-blower Attachment.

C. Edmund Kells, D.D.S., . . . . New Orleans, La.

Dental Electrics.

J. A. Swasey, D.D.S., . . . . . . . Chicago, Ill.

Dental Clamps.

We are requested to say that any dentist who has not received a direct invitation is solicited to consider himself as personally addressed by this general notice and is assured of a cordial welcome. For further particulars apply to Dr. W. W. Walker, President, 67 West Ninth street, or to Dr. A. L. Honthrop, 57 West Forty-Ninth street, New York.

### SALMAGUNDI.

### CONTENTS.

COMMUNICATIONS.

Protective Dentine, or Dentine of Repair.......... 5

Breathing Pure Oxygen.......... 17

Some Suggestions on Metal Cap Crowns.......... 17

Near Approach to, and Devitalization of the Dental Pulp.......... 23

Ammonia vs. Zinc Fillings.......... 25

Reflex(ions).......... 27

Tin and Gold as a Filling.......... 31

Extraction of Teeth.......... 32

Pyorrhœa Alveolaris.......... 35

Discussions of the Ohio State Dental Society.......... 42

First District Dental Society of New York.......... 50

SALMAGUNDI.......... 53

In Memoriam.......... 55

---

### THE DENTAL REGISTER,

**A MONTHLY MAGAZINE DEVOTED TO THE INTERESTS OF THE**

### DENTAL PROFESSION.

*Terms Reduced to* $2.00 *Per Year. Single Copies,* 25 *Cents.*

### EDITED BY PROF. J. TAFT, D.D.S.

—AND—

### Published by SAM'L A. CROCKER & CO., Ohio Dental and Surgical Depot.

The volume commences in January and closes in December.

The REGISTER being issued monthly is always fresh, therefore Indispensable to the practitioner who desires to keep posted up with the new inventions and improvements which are daily developed. Neither expense nor effort will be spared to give value and variety to its contents. Articles will be illustrated with well executed engravings whenever they will add to the interest or assist to explanation.

Its extensive circulation and frequency of issue make it one of the BEST DENTAL ADVERTISING MEDIUMS in the United States.

All subscriptions must begin with January or July.

Local or special notices, five lines or less, will be inserted for $3 each insertion; over five lines, 50 cts. per line.

All advertising bills payable in advance, or at furthest, after the issue of the first number containing the advertisement. All transient advertisements to be paid for in advance.

☞CONTRIBUTIONS SOLICITED.

Exclusively Editorial Communications should be addressed to the Editor.

The latest number of the REGISTER may be ordered with or without other goods at any time, and all letters should be addressed to

**SAM'L A. CROCKER & CO., Ohio Dental and Surgical Depot, Cincinnati, O.**

